# Rapid Improvement in Cardiac Damage Predicts Better Prognosis After Transcatheter Aortic Valve Replacement

**DOI:** 10.3390/jcdd12010029

**Published:** 2025-01-16

**Authors:** Hao-Wei Lee, Chih-Hui Chin, Po-chin Chou, Chia-Hsiu Chang, Chiu-Ling Tsai, Chi-Hung Huang

**Affiliations:** 1 General Cardiology, Cardiovascular Center, Cathay General Hospital, Taipei 106, Taiwan; howard781003@hotmail.com (H.-W.L.);; 2 School of Medicine, College of Medicine, National Yang Ming Chiao Tung University, Taipei 112, Taiwan; 3 School of Medicine, College of Medicine, Fu Jen Catholic University, New Taipei 242, Taiwan; 4 Interventional Cardiology, Cardiovascular Center, Cathay General Hospital, Taipei 106, Taiwan; 5 Division of Cardiovascular Surgery, Cardiovascular Center, Cathay General Hospital, Taipei 106, Taiwan; 6 Cardiovascular Center, Cathay General Hospital, Taipei 106, Taiwan; 7 School of Medicine, National Tsing Hua University, Hsinchu City 300, Taiwan

**Keywords:** severe aortic stenosis, cardiac damage, transcatheter aortic valve replacement

## Abstract

Background: A staging system based on cardiac damage for severe aortic stenosis (AS) has been validated for prognosis prediction following transcatheter aortic valve replacement (TAVR). Our study aims to investigate whether TAVR can lead to changes in cardiac damage shortly after the procedure and how these changes impact prognosis. Method: Patients in this retrospective cohort study were classified into five stages (0–4) before TAVR based on the echocardiographic findings of cardiac damage. The closest echocardiogram after TAVR was used for restaging cardiac damage. The primary composite outcome was all-cause mortality or hospitalization due to heart failure (HF). Results: A total of 64 patients were enrolled (53.1% male, mean age 81.7 ± 7.7 years). Within a mean interval of 4 days (interquartile range = 3 to 7 days) after TAVR, cardiac damage improved in 25.0% of patients, while it worsened in 20.3%. During a median follow-up of 2.5 ± 1.9 years, 34.4% of patients met the primary endpoint, which included 16 deaths and 6 HF hospitalizations. Cox regression analysis revealed that improvement in cardiac damage correlated with a lower risk of composite death or HF hospitalization (HR: 0.095; 95% CI: 0.014–0.627; *p* = 0.015). Conclusions: TAVR can lead to changes in cardiac damage over a short period in patients with severe AS, and rapid improvement in cardiac damage after TAVR is associated with a better prognosis.

## 1. Introduction

With the development of transcatheter aortic valve replacement (TAVR), the clinical course of patients with severe aortic stenosis (AS) has undergone significant transformation [1,2,3,4,5,6]. Still, given the reported mortality rates of up to 18% within two years post-TAVR [7], there remains a crucial need to explore clinical factors for risk stratification and prognosis associated with the procedure. Traditionally, AS patients were evaluated by focusing on symptoms and the aortic valve itself [8,9]. Instead, Généreux et al. introduced a staging system that considers both anatomic and functional cardiac damage, assessed through echocardiography, applicable to patients undergoing either surgical aortic valve replacement or TAVR [10]. Patients were classified into five stages based on the extent of cardiac damage, with its prognostic implications validated across multiple studies, revealing a graded correlation between staging and prognosis among patients with TAVR [1,11,12,13,14,15,16,17,18,19,20]. However, limited attention has been paid to the immediate changes in cardiac damage and its impact on clinical outcomes following the procedure. Thus, our study aims to investigate whether TAVR can lead to changes in cardiac damage shortly after the procedure and how these changes impact prognosis.

## 2. Materials and Methods

### 2.1. Participants

Consecutive patients with severe AS who underwent TAVR were enrolled at Cathay General Hospital in Taiwan between September 2016 and July 2024. Severe AS was defined according to current guidelines as a mean aortic valve gradient ≥ 40 mmHg and/or a peak aortic jet velocity ≥ 4 m/s and/or aortic valve area < 1.0 cm^2^ (or an indexed aortic valve area < 0.6 cm^2^/m^2^) [21,22]. Patients were excluded if they met any of the following criteria: (1) less than 6 months of follow-up after TAVR, as this period was considered too short for assessing clinical outcomes, or (2) did not receive post-procedure echocardiography.

### 2.2. Study Design

This is a retrospective, single-center cohort study. Comprehensive data were collected through a review of medical records. The study was approved by the Ethics Committee of Cathay General Hospital (approval number: CGH-P113040) and was conducted in accordance with the principles of the Declaration of Helsinki.

### 2.3. Study Procedure

**Figure 1:**
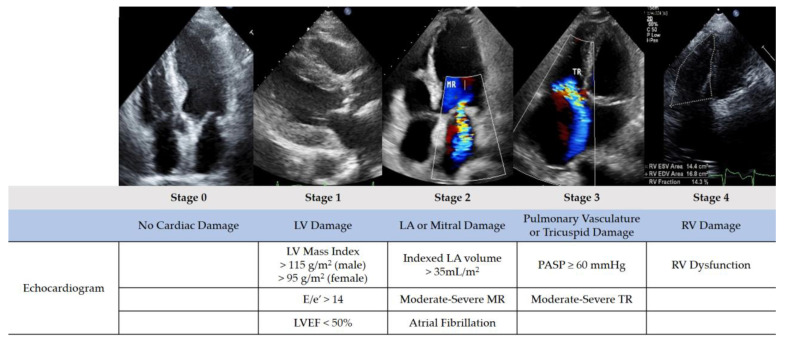
Aortic stenosis staging classification based on extent of cardiac damage. LA, left atrial; LV, left ventricular; LVEF, left ventricular ejection fraction; MR, mitral regurgitation; RV, right ventricular; PASP, pulmonary artery systolic pressure; TR, tricuspid regurgitation.

All patients in this study underwent comprehensive transthoracic echocardiography according to guidelines before TAVR and received follow-up evaluations after the procedure [23,24,25]. Each echocardiographic image and Doppler data were acquired and verified by two echocardiography specialists. During the retrospective data collection and cardiac damage staging process, each echocardiogram was independently reviewed and confirmed by two cardiologists who were blinded to the clinical information and outcome data. The stages of cardiac damage before and after TAVR were recorded, and patients were categorized into groups of improvement, no change, and worsening based on the changes in cardiac damage. Cardiac damage was classified using the same staging classification system proposed by Généreuxet et al. Patients were categorized into the following stages (Figure 1): Stage 0: no cardiac damage; Stage 1: left ventricular (LV) damage, defined by LV mass index > 115 g/m^2^ for males and > 95 g/m^2^ for females, E/e′ > 14, or left ventricular ejection fraction (LVEF) < 50%; Stage 2: left atrial (LA) or mitral valve damage, defined by the presence of dilated LA (LA volume index > 34 mL/m^2^), the presence of atrial fibrillation, or moderate or severe mitral regurgitation (MR); Stage 3: pulmonary vasculature or tricuspid damage, defined by pulmonary artery systolic pressure (PASP) ≥ 60 mmHg or the presence of moderate or severe tricuspid regurgitation (TR); and Stage 4: right ventricular (RV) damage, defined by the presence of RV dysfunction (tricuspid annular plane systolic excursion < 1.7 cm, S′ < 9.5 cm/s, or fractional area change < 35%) [10,23,24,25]. To be considered as meeting the criteria for a specific stage, a patient only needed to fulfill one of the criteria for that stage. If more than one stage of criteria is met, the patient will ultimately be classified into the highest stage that they meet the criteria for.

### 2.4. Outcomes

The primary outcome of this study was the composite of all-cause mortality or hospitalization due to heart failure (HF). Secondary outcomes included all-cause mortality, HF hospitalization, new-onset left bundle branch block (LBBB), and permanent pacemaker (PPM) implantation within 30 days.

### 2.5. Statistical Analysis

Statistical analysis was performed using the Statistical Package for Social Sciences (version 21.0, SPSS Inc., Chicago, IL, USA). All data are expressed as the mean ± standard deviation or as frequencies and percentages. Parametric continuous data were compared between different patient groups using the unpaired Student’s *t*-test, while nonparametric data were compared using the Mann–Whitney test. Categorical variables were analyzed using the chi-squared test. When cardiac damage improvement was included as a covariate, the analysis was restricted to patients in Stages 1–4 at baseline. In contrast, when cardiac damage worsening was considered as a covariate, the analysis focused on patients in Stages 0–3 at baseline. Event-free analysis was performed for the primary outcome using the Kaplan–Meier (KM) curve, with significance assessed using the log-rank test. We also conducted a Cox proportional hazards regression analysis to assess independent predictors of the primary outcome. Baseline clinical factors with a *p*-value < 0.15 in univariable models were then entered into a multivariate model. The adjusted hazard ratios (HRs) and 95% confidence intervals (CIs) were estimated after adjusting for potential confounding factors. Statistical significance was defined as a two-sided *p*-value < 0.05.

## 3. Results

### 3.1. Baseline Characteristics of Study Population

**Figure 2:**
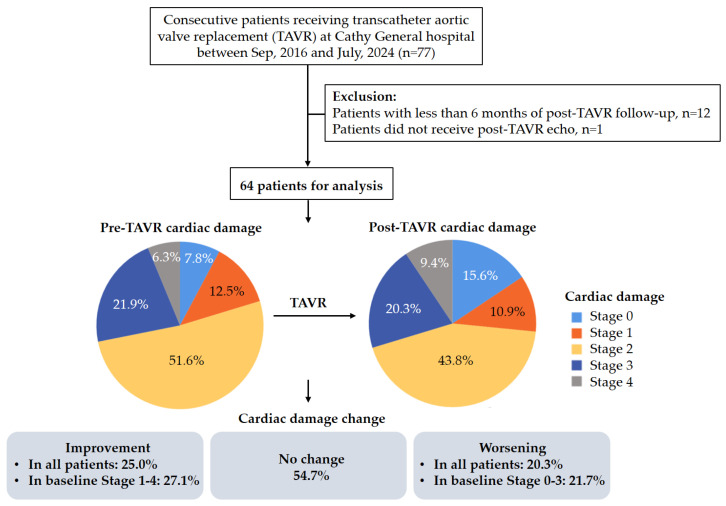
Flow chart of the study and the changes in cardiac damage classification post-TAVR.

During the study period, 77 patients received TAVR at Cathay General Hospital. A total of 13 patients were excluded based on the exclusion criteria. Consequently, the study cohort comprised a total of 64 patients suitable for analysis (Figure 2). The mean age of the participants was 81.7 ± 7.7 years, and 53.1% were men. The mean Society of Thoracic Surgeons (STS) risk score was 8.5 ± 7.0%. A total of 61 (95.3%) patients received TAVR via trans-femoral access and 3 (4.7%) patients via nontrans-femoral access. A total of 59 (92.2%) patients received a self-expanding valve, and 5 (7.8%) patients received a balloon-expandable valve. Other baseline characteristics of patients are listed in Table 1. At baseline, 5 (7.8%) patients were in Stage 0, 8 (12.5%) patients were in Stage 1, 33 (51.6%) patients were in Stage 2, 14 (21.9%) patients were in Stage 3, and 4 (6.3%) patients were in Stage 4. The mean interval between TAVR and echocardiographic follow-up after the procedure was 4 days, with an interquartile range (IQR) of 3 to 7 days, and 92.2% of the patients received evaluation during the index hospitalization.

**Figure 3:**
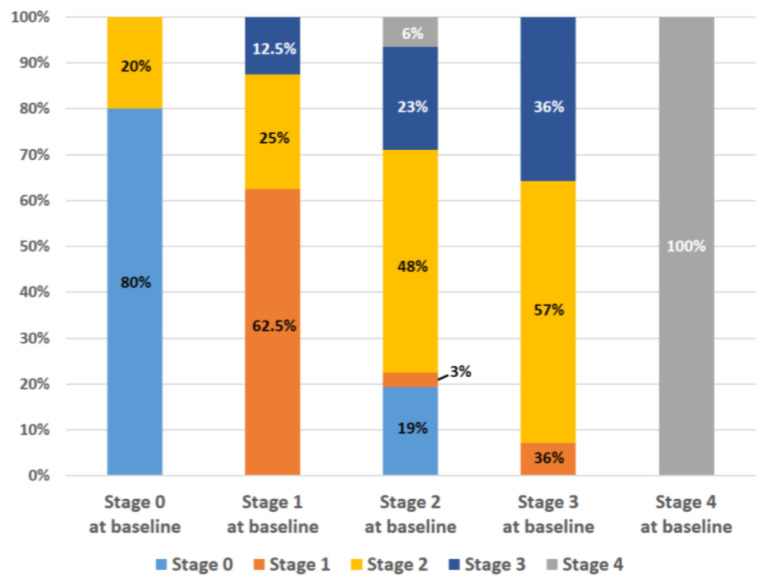
Distribution of cardiac stage post-TAVR. Each bar represents the distribution of post-TAVR stages for patients in each baseline stage.

After TAVR, 10 (15.6%) patients were in Stage 0, 7 (10.9%) patients were in Stage 1, 28 (43.8%) patients were in Stage 2, 13 (20.3%) patients were in Stage 3, and 6 (9.4%) patients were in Stage 4. The distribution of the post-TAVR cardiac stage according to the baseline stage is shown in Figure 3. A total of 16 (25.0%) patients experienced cardiac damage improvement (27.1% in baseline Stage 1–4), and 13 (20.3%) patients underwent cardiac damage worsening (21.7% in baseline Stage 0–3), while the remaining patients showed no change (Figure 2). Patients with higher SPAP (52.9 ± 13.8 vs. 41.1 ± 11.2 mmHg, *p* = 0.002) and SPAP ≥ 60 mmHg (37.5% vs. 4.7%, *p* = 0.001) were more likely to show improvement in cardiac damage after TAVR (Table A1). All patients in our study with a baseline SPAP ≥ 60 mmHg achieved improvement in pulmonary blood pressure, with post-TAVR SPAP levels falling below 60 mmHg. Additionally, 50% of patients with moderate or severe TR and 72.7% of those with moderate or severe MR at baseline experienced a reduction in valve regurgitation severity to below moderate (Table A2).

### 3.2. Outcomes

During a median follow-up of 2.5 ± 1.9 years, 22 (34.4%) patients met the primary composite endpoint of all-cause mortality or hospitalization due to HF, which included 16 (25.0%) deaths and 6 (9.4%) HF hospitalizations. Additionally, 6 (9.4%) patients had new-onset LBBB, and 5 (7.8%) patients received PPM implantation within 30 days after TAVR (Table 2). Among patients in Stage 1–4 at baseline, those with improvement in cardiac damage after TAVR were associated with a lower risk of death or HF hospitalization compared to those without improvement (12.5% vs. 44.2%, *p* = 0.024). On the other hand, among patients in Stage 0–3, no significant association was found between worsening cardiac damage and the primary outcome (30.8% vs. 34.0%, *p* = 0.825) (Table 2). Furthermore, neither cardiac damage improvement nor worsening showed a significant association with the various secondary outcomes (Table 2).

**Figure 4:**
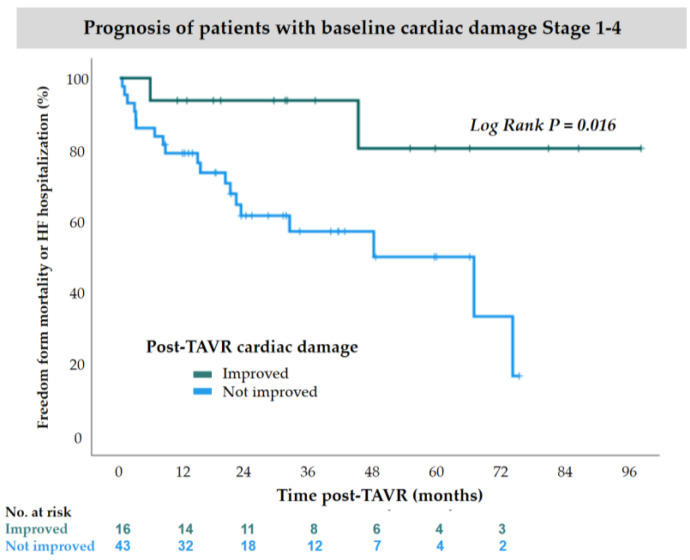
Kaplan–Meier survival curves for mortality or heart failure hospitalization in patients with cardiac damage improved after TAVR vs. cardiac damage not improved after TAVR (*p* = 0.016, log-rank test) among Stage 1–4 patients. HF, heart failure; TAVR, transcatheter aortic valve replacement.

**Figure A1:**
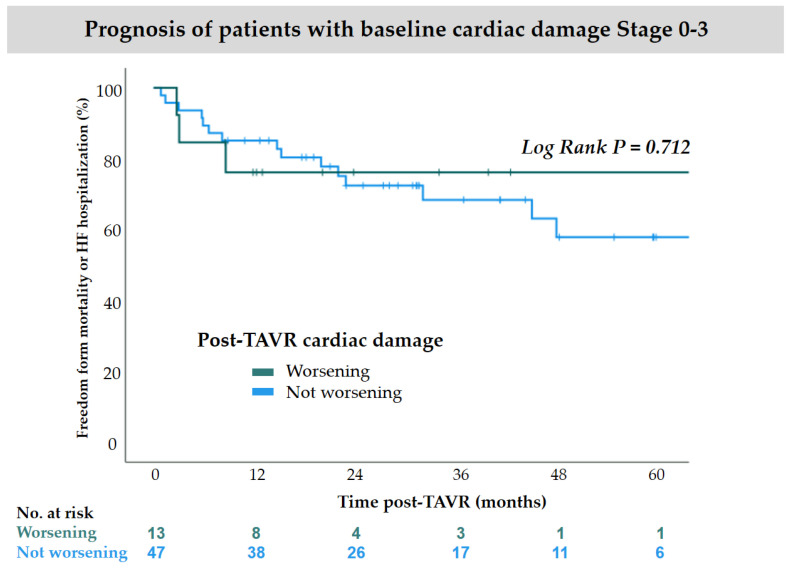
Kaplan–Meier survival curves for mortality or heart failure hospitalization in patients with cardiac damage improved after TAVR vs. cardiac damage not improved after TAVR (*p* = 0.712, log-rank test) among Stage 0–3 patients. HF, heart failure; TAVR, transcatheter aortic valve replacement.

Among patients in Stage 1–4 at baseline, the KM curve showed a lower event rate of mortality or HF hospitalization in patients with cardiac damage improvement after TAVR, compared to those without improvement (log-rank *p* = 0.016) (Figure 4), whereas cardiac damage worsening did not significantly affect clinical outcomes in Stage 0–3 patients (Figure A1). In univariate analyses, among patients in Stage 1–4 at baseline, older age was associated with a higher rate of the composite outcome of mortality or HF hospitalization (HR: 1.152; 95% CI: 1.043–1.272; *p* = 0.005), while higher body mass index and cardiac damage improvement were associated with lower event rates (HR: 0.788; 95% CI: 0.646–0.961; *p* = 0.018 and HR: 0.180; 95% CI: 0.036–0.893; *p* = 0.036). Covariates including age, body mass index, diabetes mellitus, chronic kidney disease, and cardiac damage improvement were entered into a multivariate analysis. The multivariate model demonstrated that cardiac damage improvement was independently associated with a lower risk of the composite outcome of death or hospitalization due to HF (HR: 0.095; 95% CI: 0.014–0.627; *p* = 0.015). Additionally, older age was associated with a poorer prognosis (HR: 1.139; 95% CI: 1.021–1.270; *p* = 0.019) (Table 3).

## 4. Discussion

The main findings of the study were as follows: (1) TAVR can modify the extent of cardiac damage in patients with severe AS within a short time; (2) patients with elevated SPAP were more likely to experience improvement in cardiac damage after TAVR, highlighting the procedure’s ability to alleviate right heart pressure; and (3) improvement in cardiac damage shortly after TAVR was significantly associated with a lower risk of the composite outcome of death or HF hospitalization.

The staging system for cardiac damage introduced by Généreux et al. demonstrated a strong graded association between the extent of cardiac damage and post-TAVR prognosis, and it has been well validated in many TAVR trials [1,10,11,12,13,14,15,16,17,18,19,20,26]. However, there are only a few studies discussing the changes in cardiac damage and their influence on clinical outcomes after TAVR. Using data from the PARTNER (Placement of Aortic Transcatheter Valves) 2 and 3 trials, including 1179 patients who underwent TAVR, Généreux et al. found that 15.6% of patients experienced improvement, while 26.5% showed worsening in cardiac damage. Furthermore, the changes in cardiac damage stages at 1 year were independently associated with mortality (adjusted HR for improvement: 0.49; worsening: 1.95; *p* = 0.023) and the composite outcome of death or HF hospitalization (adjusted HR for improvement: 0.60; worsening: 2.25; *p* < 0.001) at 2 years [26]. Another Swiss single-center study examined cardiac damage changes in 1863 patients within a much shorter interval after TAVR, with 98.2% assessed at discharge and 1.9% at 30 days post-TAVR. The study revealed that the cardiac stage improved in 30.1% of patients in Stage 1–4 and deteriorated in 24.7% of patients in Stage 0–3. Among patients in Stage 2–4, 5-year all-cause mortality was stratified by changes in cardiac damage (improved, unchanged, or worsened) (log-rank *p* < 0.001 for Stage 2, 0.005 for Stage 3, and <0.001 for Stage 4) [27]. At the same time, there are significant differences between Asian and Western populations in terms of clinical and anatomic features. A lower body mass index, a smaller annulus area, a smaller sinus of Valsalva, and a lower height of the coronary ostium were reported among Asian patients receiving TAVR, which may remarkably affect the treatment strategy and the risks related to TAVR [28,29]. Still, studies focused on the Asian population when exploring the change in cardiac damage are limited. A Chinese single-center study involving 644 patients compared the stages of cardiac damage within 30 days after TAVR, revealing that cardiac damage had changed in 22.2% of patients, and the deterioration in cardiac damage was an independent risk factor for 2-year mortality (HR:19.564; 95% CI: 8.047–47.565; *p* < 0.001) [30]. In our study, 45.3% of patients exhibited changes in cardiac damage within a median of 4 days (IQR = 3 to 7 days) after TAVR, which is a much shorter interval compared to those in previous studies [26,30]. Moreover, as the PARTNER 2 and 3 trials only involved intermediate- and low-risk patients, respectively [26], and the median STS risk scores of the Swiss (5.4 ± 3.9%) and Chinese (2.8 [1.9–5.1] % in Stage 0, 5.1 [3.4–8.4] % in Stage 1, 4.4 [2.8–8.0] % in Stage 2, and 5.9 [4.1–9.3] % in Stage 3) cohort studies were relatively low [27,30], the patients in our study presented with a higher STS risk score of 8.5 ± 7.0%. Our study validated the rapid impact that TAVR can have on cardiac alterations and its correlation with clinical outcomes in an Asian population with relatively high surgical risk.

Our study shows that TAVR can alter cardiac damage in patients with severe AS, with improvements linked to better prognosis. However, a recent single-center observational study suggests that TAVR may not fully reverse LV myocardial damage [31], highlighting the importance of addressing cardiac damage through adjunctive medical therapies. An international registry of 311 diabetic patients undergoing TAVR found that sodium-glucose cotransporter-2 inhibitors (SGLT2i) were associated with better clinical outcomes and influenced cardiac remodeling. Among SGLT2i users, 92.7% exhibited stable or improved cardiac damage within 1 year, compared to 21.3% of non-users who progressed to worse stages, reflecting on the greater increase in LVEF and reduction in PASP in the SGLT2i group [32]. Given the limited evidence, further research is essential to explore the role of pharmacological treatments in cardiac remodeling post-TAVR, aiming to optimize management strategies for severe AS patients.

In our study, patients with pulmonary hypertension (PH) demonstrated a greater potential for improvement in cardiac damage. PH often coexists with AS and has been shown to be a predictor of mortality following TAVR. Among the subtypes of PH, combined post- and pre-capillary PH (combined PH) exhibited a strong correlation with poor survival. O’Sullivan et al. published a retrospective study involving 606 patients, revealing a higher 1-year mortality rate after TAVR among those with combined PH compared to those without PH (HR: 3.15; 95% CI: 1.43–6.93; *p* = 0.004) [33]. Similarly, in another retrospective study of 503 TAVR patients by Lukas Weber et al., combined PH was identified as an independent predictor of death (HR: 4.39; 95% CI: 2.40–8.03; *p* < 0.001) [34]. In contrast, both studies indicated that patients with isolated postcapillary PH had a similar survival rate post-TAVR compared to patients without PH [33,34]. In cases of isolated postcapillary PH, the elevation of SPAP is believed to result from the passive backward transmission of the increased LV filling pressure related to severe AS. Therefore, the elevated pressure in the right heart is considered reversible in isolated postcapillary PH after TAVR. An immediate improvement in SPAP was also observed via echocardiogram after TAVR among patients with postcapillary PH in the study by O’Sullivan et al. (50.2 ± 13.7 mmHg vs. 44.9 ± 14.3 mmHg; *p* = 0.001) [33]. Notably, all patients with a baseline SPAP ≥ 60 mmHg in our study experienced a reduction in pulmonary pressure, confirming that the reversibility of elevated SPAP is achievable through TAVR and suggesting that a specific subset of PH patients may benefit from this intervention.

Our study offers several key contributions. It is one of the few to examine the impact of cardiac damage changes on TAVR prognosis. We also provide insights into early post-operative assessments of cardiac damage, with evaluations conducted relatively soon after the procedure, which may provide additional perspectives compared to previous studies. Additionally, we focus on an Asian cohort, which remains underrepresented when discussing cardiac damage change in TAVR research, potentially offering valuable regional insights. Lastly, by including patients with higher surgical risk, our study helps enhance understanding of TAVR outcomes in this more complex population.

This study has several limitations. First, it was a single-center retrospective study with a small population, and further research with larger sample sizes is needed to warrant our results. Second, the echocardiographic parameters of E/e’ were not completely captured, hindering us from applying the staging system. Third, the echocardiographic parameters used for cardiac damage staging are potentially subject to variability and measurement errors. Fourth, our patients did not receive post-TAVR echocardiography within a uniform timeframe, and the cardiac damage may be influenced by the variability in the time window. Finally, most patients received a self-expanding valve, with only a small number receiving a balloon-expandable valve. This may affect the generalizability of our findings.

## 5. Conclusions

TAVR can lead to changes in cardiac damage in patients with severe AS over a short period, and rapid improvement in cardiac damage after TAVR is associated with a lower risk of the composite of death or HF hospitalization. Our study also suggests that a specific subgroup of patients with PH is potentially more likely to experience improvement in cardiac damage after TAVR.

## Figures and Tables

**Table 1:** Baseline characteristics.

	All (*n* = 64)	Stage 0 (*n* = 5)	Stage 1 (*n* = 8)	Stage 2 (*n* = 33)	Stage 3 (*n* = 14)	Stage 4 (*n* = 4)	*p*-Value
Age, years	81.7 ± 7.7	82.6 ± 5.9	79.0 ± 7.8	81.7 ± 7.3	81.6 ± 9.0	86.0 ± 9.6	0.692
Male, *n* (%)	34 (53.1%)	4 (80.0%)	5 (62.5%)	18 (54.5%)	5 (35.7%)	2 (50.0%)	0.481
Body mass index, kg/m^2^	24.7 ± 3.6	25.3 ± 2.9	23.4 ± 2.8	25.2 ± 3.8	24.3 ± 3.9	24.2 ± 2.6	0.711
Hypertension, *n* (%)	57 (89.1%)	4 (80.0%)	7 (87.5%)	29 (87.9%)	13 (92.9%)	4 (100.0%)	0.880
Diabetes mellitus, *n* (%)	35 (54.7%)	1 (20.0%)	5 (62.5%)	18 (54.5%)	9 (64.3%)	2 (50.0%)	0.528
Heart failure, *n* (%)	32 (50.0%)	1 (20.0%)	2 (25.0%)	14 (42.4%)	11 (78.6%)	4 (100.0%)	0.011
Atrial fibrillation, *n* (%)	16 (25.0%)	0 (0.0%)	0 (0.0%)	7 (21.2%)	7 (50.0%)	2 (50.0%)	0.032
Coronary artery disease, *n* (%)	33 (51.6%)	2 (40.0%)	4 (50.0%)	15 (45.5%)	8 (57.1%)	4 (100.0%)	0.319
Triple vessel ± LM disease, *n* (%)	14 (21.9%)	1 (20.0%)	2 (25.0%)	6 (18.2%)	4 (28.6%)	1 (25.0%)	0.950
Coronary artery bypass graft, *n* (%)	7 (10.9%)	1 (20.0%)	1 (12.5%)	3 (9.1%)	2 (14.3%)	0 (0.0%)	0.877
Cerebrovascular disease, *n* (%)	5 (7.8%)	0 (0.0%)	1 (12.5%)	3 (9.1%)	1 (7.1%)	0 (0.0%)	0.896
PAOD, *n* (%)	14 (21.9%)	1 (20.0%)	4 (50.0%)	8 (24.2%)	1 (7.1%)	0 (0.0%)	0.151
Chronic kidney disease, *n* (%)	37 (57.8%)	3 (60.0%)	5 (62.5%)	17 (51.5%)	9 (64.3%)	3 (75.0%)	0.854
Asthma or COPD, *n* (%)	8 (12.5%)	2 (40.0%)	2 (25.0%)	3 (9.1%)	1 (7.1%)	0 (0.0%)	0.208
Prior permanent pacemaker, *n* (%)	8 (12.5%)	0 (0.0%)	0 (0.0%)	5 (15.2%)	1 (7.1%)	2 (50.0%)	0.108
STS risk score, %	8.5 ± 7.0	3.4 ± 1.8	9.5 ± 5.3	6.4 ± 4.8	13.6 ± 8.8	12.2 ± 11.7	0.004
**Echocardiographic findings**							
LVEF, %	58.9 ± 14.4	67.2 ± 4.2	56.5 ± 20.2	62.2 ± 11.8	52.9 ± 14.7	47.8 ± 16.7	0.070
AV peak velocity, m/s	4.1 ± 0.9	3.9 ± 0.5	4.4 ± 0.9	4.2 ± 1.0	3.9 ± 0.6	3.3 ± 0.6	0.149
AV MPG, mmHg	40.5 ± 18.4	37.0 ± 11.1	45.6 ± 18.6	44.4 ± 21.0	34.1 ± 11.8	25.3 ± 9.4	0.150
Moderate or severe MR, *n* (%)	11 (17.2%)	0 (0.0%)	1 (12.5%)	5 (15.2%)	4 (28.6%)	1 (25.0%)	0.609
Moderate or severe TR, *n* (%)	10 (15.6%)	0 (0.0%)	0 (0.0%)	0 (0.0%)	9 (64.3%)	1 (25.0%)	<0.001
PASP ≥ 60 mmHg, *n* (%)	8 (12.5%)	0 (0.0%)	0 (0.0%)	0 (0.0%)	7 (50.0%)	1 (25.0%)	<0.001
PASP, mmHg	43.0 ± 13.0 ^1^	29.2 ± 2.2	37.4 ± 7.0	39.5 ± 10.3 ^2^	58.0 ± 10.0	46.0 ± 15.2	<0.001
**Procedural findings**							
Nontrans-femoral access, *n* (%)	3 (4.7%)	0 (0.0%)	0 (0.0%)	1 (3.0%)	0 (0.0%)	2 (50.0%)	<0.001
Self-expanding valve, *n* (%)	59 (92.2%)	5 (100.0%)	7 (87.5%)	32 (97.0%)	11 (78.6%)	4 (100.0%)	0.226
Balloon-expandable valve, *n* (%)	5 (7.8%)	0 (0.0%)	1 (12.5%)	1 (3.0%)	3 (21.4%)	0 (0.0%)	0.226

AV, aortic valve; COPD, chronic obstructive pulmonary disease; LM, left main; LVEF, left ventricular ejection fraction; MPG, mean pressure gradient; MR, mitral regurgitation; PAOD, peripheral arterial occlusion disease; PASP, pulmonary artery systolic pressure; STS, Society of Thoracic Surgeons; TR, tricuspid regurgitation; ^1^ *n* = 61; ^2^ *n* = 30.

**Table 2:** Clinical outcomes of all patients and according to cardiac damage change.

		Cardiac Damage Change					
		Stage 1–4 (*n* = 59)			Stage 0–3 (*n* = 60)		
	All (*n* = 64)	Improved (*n* = 16)	Not Improved (*n* = 43)	*p*-Value	Worsening (*n* = 13)	Not Worsening (*n* = 47)	*p*-Value
**Primary outcome**							
Mortality or HF hospitalization, *n* (%)	22 (34.4%)	2 (12.5%)	19 (44.2%)	0.024	4 (30.8%)	16 (34.0%)	0.825
**Secondary outcomes**							
Mortality, *n* (%)	16 (25.0%)	2 (12.5%)	13 (30.2%)	0.164	3 (23.1%)	12 (25.5%)	0.856
HF hospitalization, *n* (%)	6 (9.4%)	0 (0.0%)	6 (14.0%)	0.115	1 (7.7%)	4 (8.5%)	0.925
New-onset LBBB, *n* (%)	6 (9.4%)	1 (6.3%)	5 (11.6%)	0.543	0 (0.0%)	5 (10.6%)	0.219
PPM implantation ^1^, *n* (%)	5 (7.8%)	0 (0.0%)	4 (9.3%)	0.206	1 (7.7%)	4 (8.5%)	0.925

HF, heart failure; LBBB, left bundle branch block; PPM, permanent pacemaker; ^1^ within 30 days after TAVR.

**Table 3:** Univariable and multivariable Cox proportional hazards analysis on mortality or heart failure hospitalization.

	Univariable		Multivariable	
	HR (95% CI)	*p*-Value	HR (95% CI)	*p*-Value
Age, years	1.152 (1.043–1.272)	0.005	1.139 (1.021–1.270)	0.019
Male	1.100 (0.379–3.196)	0.861		
Body mass index, kg/m^2^	0.788 (0.646–0.961)	0.018	0.804 (0.625–1.033)	0.088
Hypertension	0.514 (0.094–2.810)	0.443		
Diabetes mellitus	0.390 (0.131–1.164)	0.092	0.277 (0.058–1.332)	0.109
Heart failure	1.333 (0.456–3.899)	0.599		
Atrial fibrillation	0.767 (0.225–2.610)	0.671		
Coronary artery disease	0.736 (0.253–2.143)	0.574		
Triple vessel ± LM disease	0.758 (0.202–2.842)	0.681		
CABG	0.330 (0.036–3.031)	0.327		
Cerebrovascular disease	1.228 (0.188–8.003)	0.830		
PAOD	1.172 (0.329–4.179)	0.807		
Chronic kidney disease	2.500 (0.799–7.821)	0.115	2.806 (0.637–12.360)	0.173
Asthma or COPD	1.172 (0.329–4.179)	0.807		
Prior permanent pacemaker	1.100 (0.235–5.143)	0.904		
STS risk score, %	1.025 (0.952–1.104)	0.512		
LVEF, %	1.009 (0.972–1.047)	0.645		
AV peak velocity, m/s	0.868 (0.482–1.564)	0.638		
AV MPG, mmHg	1.005 (0.977–1.034)	0.744		
Moderate or severe MR	1.667 (0.441–6.301)	0.452		
Moderate or severe TR	1.255 (0.311–5.065)	0.750		
PASP ≥ 60 mmHg	1.100 (0.235–5.143)	0.904		
Nontrans-femoral access	0.900 (0.077–10.554)	0.933		
Self-expanding valve	0.814 (0.125–5.306)	0.830		
Cardiac damage improved	0.180 (0.036–0.893)	0.036	0.095 (0.014–0.627)	0.015

AV, aortic valve; CABG, coronary artery bypass graft; CI: confidence interval; COPD, chronic obstructive pulmonary disease; HR, hazard ratio; LM, left main; MR, mitral regurgitation; PAOD, peripheral arterial occlusion disease; PASP, pulmonary artery systolic pressure; STS, Society of Thoracic Surgeons; TR, tricuspid regurgitation.

## Data Availability

The data that support the findings of this study are not publicly available due to containing information that could compromise the privacy of research participants but are available from the corresponding author upon reasonable request.

## Appendix A

**Table A1:** Baseline characteristics based on cardiac damage improvement after TAVR among patients in Stage 1–4.

Baseline Characteristics	Cardiac Damage Improved (*n* = 16)	Cardiac Damage Not Improved (*n* = 43)	*p*-Value
Age, years	80.8 ± 8.3	81.9 ± 7.8	0.626
Male, *n* (%)	9 (56.3%)	21 (48.8%)	0.613
Body mass index, kg/m^2^	24.6 ± 3.3	24.7 ± 3.8	0.925
Hypertension, *n* (%)	13 (81.3%)	40 (90.3%)	0.183
Diabetes mellitus, *n* (%)	7 (43.8%)	27 (62.8%)	0.188
Heart failure, *n* (%)	10 (62.5%)	21 (48.8%)	0.350
Atrial fibrillation, *n* (%)	4 (25.0%)	12 (27.9%)	0.823
Coronary artery disease, *n* (%)	9 (56.3%)	22 (51.2%)	0.728
Triple vessel ± left main disease, *n* (%)	4 (25.0%)	9 (20.9%)	0.737
Coronary artery bypass graft, *n* (%)	2 (12.5%)	4 (9.3%)	0.718
Cerebrovascular disease, *n* (%)	0 (0.0%)	5 (11.6%)	0.154
PAOD, *n* (%)	3 (18.8%)	10 (23.3%)	0.710
Chronic kidney disease, *n* (%)	7 (43.8%)	27 (62.8%)	0.188
Asthma or COPD, *n* (%)	2 (12.5%)	4 (9.3%)	0.718
Prior permanent pacemaker, *n* (%)	2 (12.5%)	6 (14.0%)	0.885
STS risk score, %	9.0 ± 7.9	8.9 ± 6.8	0.955
**Echocardiographic findings**			
Left ventricular ejection fraction, %	55.6 ± 13.6	59.2 ± 15.1	0.416
AV peak velocity, m/s	4.4 ± 0.6	4.0 ± 1.0	0.054
AV mean pressure gradient, mmHg	44.8 ± 13.4	39.4 ± 20.1	0.248
Moderate or severe MR, *n* (%)	1 (6.3%)	10 (23.3%)	0.136
Moderate or severe TR, *n* (%)	4 (25.0%)	6 (14.0%)	0.315
PASP ≥ 60 mmHg, *n* (%)	6 (37.5%)	2 (4.7%)	0.001
PASP, mmHg	52.9 ± 13.8 ^1^	41.1 ± 11.2 ^2^	0.002
**Procedural findings**			
Nontrans-femoral access, *n* (%)	0 (0.0%)	3 (7.0%)	0.278
Self-expanding valve, *n* (%)	14 (87.5%)	40 (93.0%)	0.498
Balloon-expandable valve, *n* (%)	2 (12.5%)	3 (7.0%)	0.498

AV, aortic valve; COPD, chronic obstructive pulmonary disease; MR, mitral regurgitation; PAOD, peripheral arterial occlusion disease; PASP, pulmonary artery systolic pressure; STS, Society of Thoracic Surgeons; TAVR, transcatheter aortic valve replacement; TR, tricuspid regurgitation; ^1^ *n* = 15; ^2^ *n* = 41.

**Table A2:** Evolution in each stage’s components at post-TAVR compared with baseline.

			Post-TAVR Change	
	Baseline	Post-TAVR	Abnormal at Baseline and Normalized After TAVR	Normal at Baseline and Worsened After TAVR
Stage 1				
Increased LV mass index	16/64 (25.0%)	16/64 (25.0%)	5/16 (31.3%)	5/48 (10.4%)
E/e′ > 14	27/41 (42.2%)	26/41 (40.6%)	3/18 (16.7%)	3/12 (25%)
LVEF < 50%	15/64 (23.4%)	10/64 (15.6%)	5/15 (33.3%)	0/49 (0.0%)
Stage 2				
LAVi > 34 mL/m^2^	47/64 (73.4%)	44/64 (68.6%)	9/47 (19.1%)	6/17 (35.3%)
Atrial fibrillation	16/64 (25.0%)	16/64 (25.0%)	0/16 (0.0%)	0/48 (0.0%)
Moderate/Severe MR	11/64 (17.2%)	5/64 (7.8%)	8/11 (72.7%)	2/53 (3.8%)
Stage 3				
PASP ≥ 60 mmHg	8/64 (12.5%)	6/64 (9.4%)	8/8 (100.0%)	6/56 (10.7%)
Moderate/Severe TR	10/64 (15.6%)	12/64 (18.8%)	5/10 (50.0%)	7/54 (13.0%)
Stage 4				
RV dysfunction	4/64 (6.3%)	6/64 (9.4%)	0/4 (0.0%)	2/60 (3.3%)

LAVi, left atrial volume index; LV, left ventricular; LVEF, left ventricular ejection fraction; MR, mitral regurgitation; RV, right ventricular; PASP, pulmonary artery systolic pressure; TAVR, transcatheter aortic valve replacement; TR, tricuspid regurgitation.
